# Cage-like microstructures via sequential Ugi reactions in aqueous emulsions

**DOI:** 10.3762/bjoc.20.179

**Published:** 2024-08-22

**Authors:** Rita S Alqubelat, Yaroslava A Menzorova, Maxim A Mironov

**Affiliations:** 1 Department of Technology for Organic Synthesis, Ural Federal University, Mira St. 19, Ekaterinburg, 620002, Russian Federation

**Keywords:** carboxymethylcellulose, chitosan, colloidosomes, Pickering emulsions, Ugi reaction

## Abstract

Cage-like microstructures were obtained in two steps by sequential Ugi reactions. At the first stage, submicron colloidal particles based on carboxymethylcellulose and chitosan with a domain structure were obtained in an aqueous suspension. In the second stage, the Ugi reaction was carried out on the surface of the Pickering emulsions with toluene. Removal of toluene and redissolution in water resulted in colloidosomes with large holes on the surface. Varying the cross-link density during the Ugi reaction made it possible to obtain structures with different hole sizes.

## Introduction

Colloidosomes are microcapsules built on the basis of colloidal particles [1]. In the past 20 years, many methods have been proposed for the synthesis based on different particle types (inorganic, polymer, and composite) [2–4] and shape [5] (spheres, discs, and fibers). A distinctive feature of colloidosomes is the presence of pores, the sizes of which depend on the parameters of the original colloidal particles and the synthetic method [6]. Pores provide controlled permeability of these structures and open up rich opportunities for practical uses as catalysts, sorbents, and carriers of medicinal substances [7]. A particularly interesting area is the use of colloidosomes to model processes in living cells and produce artificial protocells [8–9]. The enlargement of pores on the surface of colloidosomes leads to structures that resemble microscopic cages [10]. Obtaining such cage-like structures is usually more complex than conventional colloidosomes and involves several stages. Pickering emulsions or polymer spheres coated with a monolayer of colloidal particles are used as initial templates for synthesis [11].

Thus, cage-like structures have been obtained using phase inversion in Pickering emulsions. The polystyrene particles were treated with sulfuric acid to form a surface layer of sulfonated polystyrene. These particles were then used to produce Pickering emulsions, which were transformed into large-pore structures when treated with an alcohol/water mixture [12]. Another method involves the formation of a double Pickering emulsion using a microfluidic technique. Solidification of this structure and removal of the template resulted in the formation of nonspherical capsules with large openings [13]. The use of a polymer matrix that can swell in an organic solvent made it possible to obtain colloidosomes covered with fissures of a controlled size [10]. A simpler method recently proposed involves the self-assembly of starch granules on the surface of emulsion droplets. However, this approach did not allow the pore size to be controlled [14]. Thus, the currently available methods for obtaining cage-like structures are characterized by technical complexity, a multi-stage nature, and low yield of target structures.

To develop a one-step method for the synthesis of cage-like structures, it is necessary to control the deposition process of colloidal particles on the surface of emulsion droplets. As a rule, spherical particles of the same size form a uniform layer, one particle thick [5]. The soft microgel particles can deform when adsorbed onto the droplet surface and form a shield-like surface [15]. Nonspherical particles can form more complex 2D structures at the interface [16]. However, the relationship between the structure of the initial particles and the surface morphology of colloidosomes is not yet understood clearly enough. Quite often, there are cases where the number of colloidal particles is clearly insufficient to cover the surface of the droplets observed in stable Pickering emulsions. We also recently encountered this problem when we obtained Pickering emulsions stabilized by cellulose particles with an extremely low concentration, which would not have been enough to form a monolayer at the interface [17]. We assumed that in this case, a cage-like structure was formed on the surface of the oil droplets, such that the material was consumed more economically.

In this study, we present preliminary data that supports this assumption. Nonspherical particles with a raspberry-like domain structure spontaneously formed a cage-like structure on the surface of emulsion droplets. Fixing this structure with a cross-linker opened the way to the one-step synthesis of colloidosomes with large pores on the surface.

## Results and Discussion

Previously, we have obtained stable colloidal particles by ionic gelation of carboxymethylcellulose (CMC) and low-molecular-weight chitosan in dilute aqueous solutions [17]. The use of this method led to the formation of particles with an average diameter in the range of 200–350 nm and with a domain structure. Domains of about 40 nm in size, consisting of CMC, were connected to each other by a soft material based on chitosan. Such structures existed only within a narrow pH range: when the pH value dropped below 5.5, the chitosan easily dissolved, and when the pH value was above 7, the CMC domains swelled and lost their properties. It is known that the Ugi reaction can be successfully used to obtain cross-linked gels based on polysaccharides [18]. Therefore, to stabilize such structures, the Ugi reaction in water was chosen, for which this particular pH value range is optimal [19]. The amino groups of chitosan and the carboxy groups of CMC reacted with each other and formed a stable structure that was no longer sensitive to changes in pH value. According to atomic force microscopy (AFM) data, the resulting particles had a domain structure and were similar in appearance to raspberries [20]. An interesting feature of the resulting particles was the high efficiency as Pickering emulsifiers: even at a concentration below 0.3 g/L, stable emulsions with linseed oil were obtained [17].

Analyzing the results obtained, we came to the conclusion that at a concentration of 0.3 g/L and with an average size of emulsion droplets of about 5 μm, the number of particles was insufficient to completely cover the surface of the droplets. In principle, two possible mechanisms were considered: (1) Soft CMC/chitosan gel particles interacted with the surface and flattened to form discs. (2) When particles were deposited on the surface, they did not completely cover it, forming a network. In this work, we tested these hypotheses and found arguments in favor of the second mechanism.

**Scheme 1:**
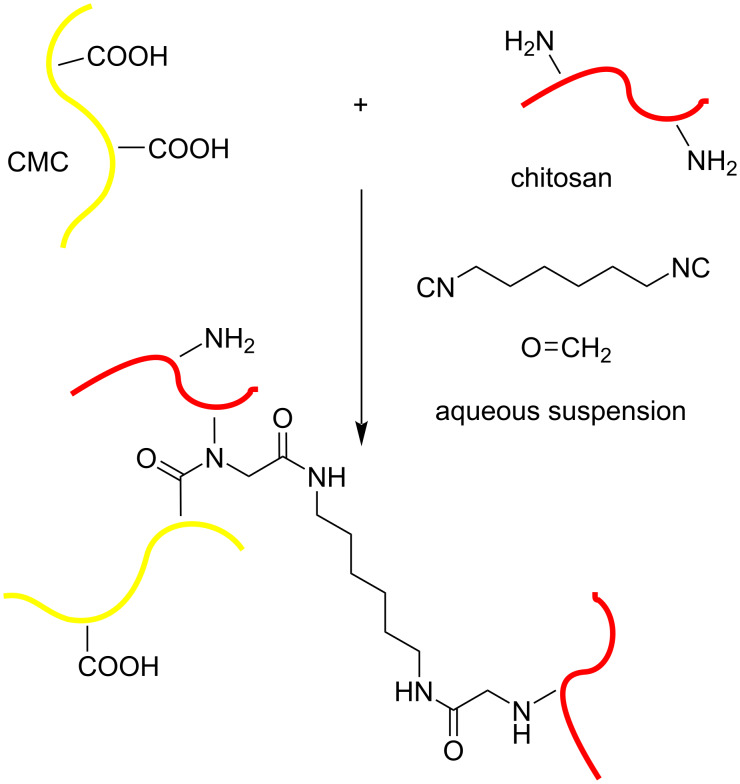
Synthesis of cross-linked microgel labelled with aminofluorescein.

To identify the structure obtained on the surface of the droplets during the formation of the Pickering emulsion, we used fluorescent labels. Aminofluorescein was introduced into the CMC composition with a ratio of 3 mg per 1 g of cellulose using condensation in the presence of dicyclohexylcarbodiimide in dimethylformamide. The resulting product was precipitated with acetonitrile, dried, and redissolved in water. Fluorescein-labelled cellulose was used to produce submicron particles according to our previously described method via the Ugi reaction in water (Scheme 1) [17].

**Figure 1:**
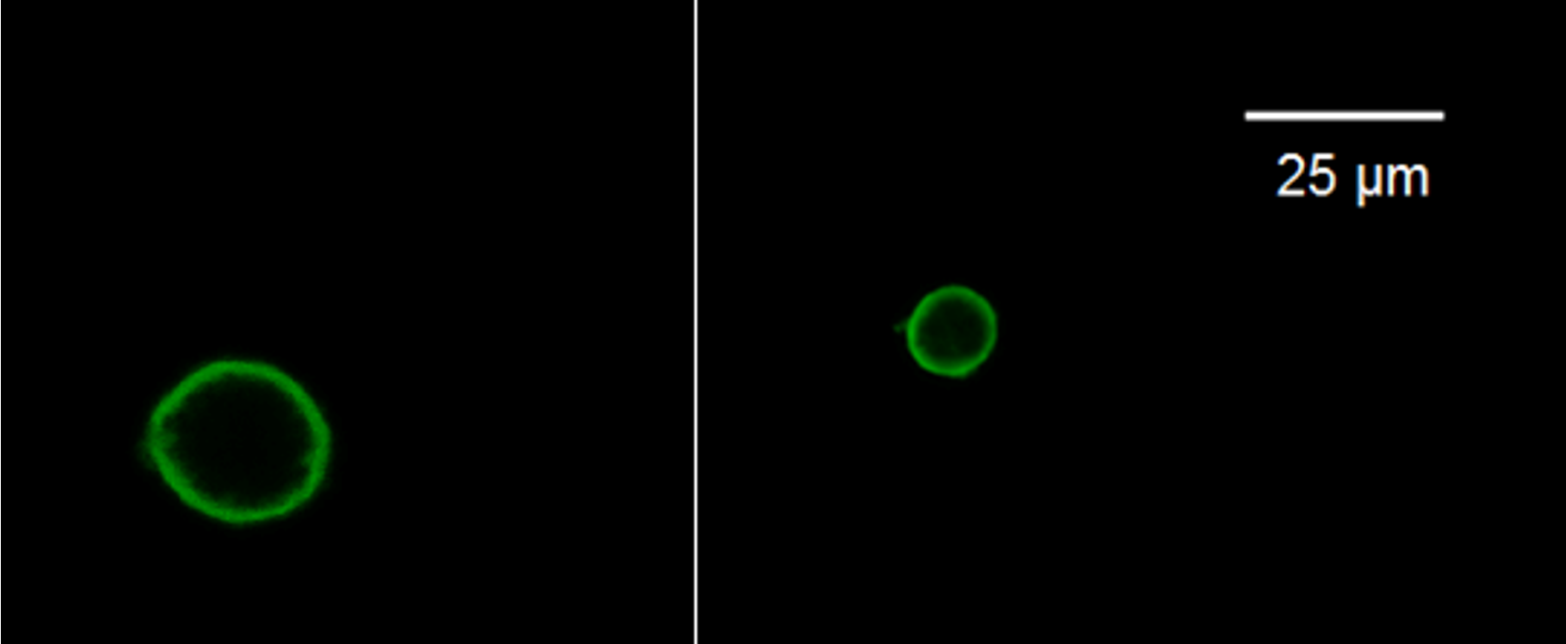
Single droplets of the Pickering emulsion attached to a glass surface.

As before, depending on the CMC concentration, we obtained submicron particles with an average diameter from 200–600 nm, which were visible under an optical microscope in the form of luminous points. Next, Pickering emulsions were obtained by emulsifying toluene in an aqueous suspension of particles with a concentration of 0.3 g/L and a water/toluene ratio of 3:1. However, it was not possible to distinguish any details on the surface of the droplets using conventional and confocal microscopy (Figure 1). To do this, it was necessary to remove the solvent that interfered with obtaining a contrast image. In turn, removal of the solvent was possible only after cross-linking since otherwise, the structure would have collapsed upon drying.

Therefore, a second Ugi reaction was carried out after the particles had been deposited on the surface of the toluene droplets. Thus, we carried out sequential Ugi reactions, the first to form colloidal particles and the second to consolidate the Pickering emulsion structure. In both cases, the same technique was used: a mixture of hexamethylene diisocyanide and formalin in acetonitrile was added dropwise into an aqueous suspension or emulsion at a ratio of 1:10 relative to the number of moles of amino groups in the chitosan. For the Ugi reaction to proceed to completion in water, it was ensured that the pH value did rise above 5.5. It should be noted that the reaction proceeded very quickly under these conditions, being completed within 2–3 min, which was monitored through the disappearance of isocyanide in the reaction medium using a color reaction [19]. Then the emulsion was applied to the glass surface and evaporated at a temperature of 50 °C. The resulting samples were analyzed using fluorescence microscopy at a wavelength of 490 nm.

**Figure 2:**
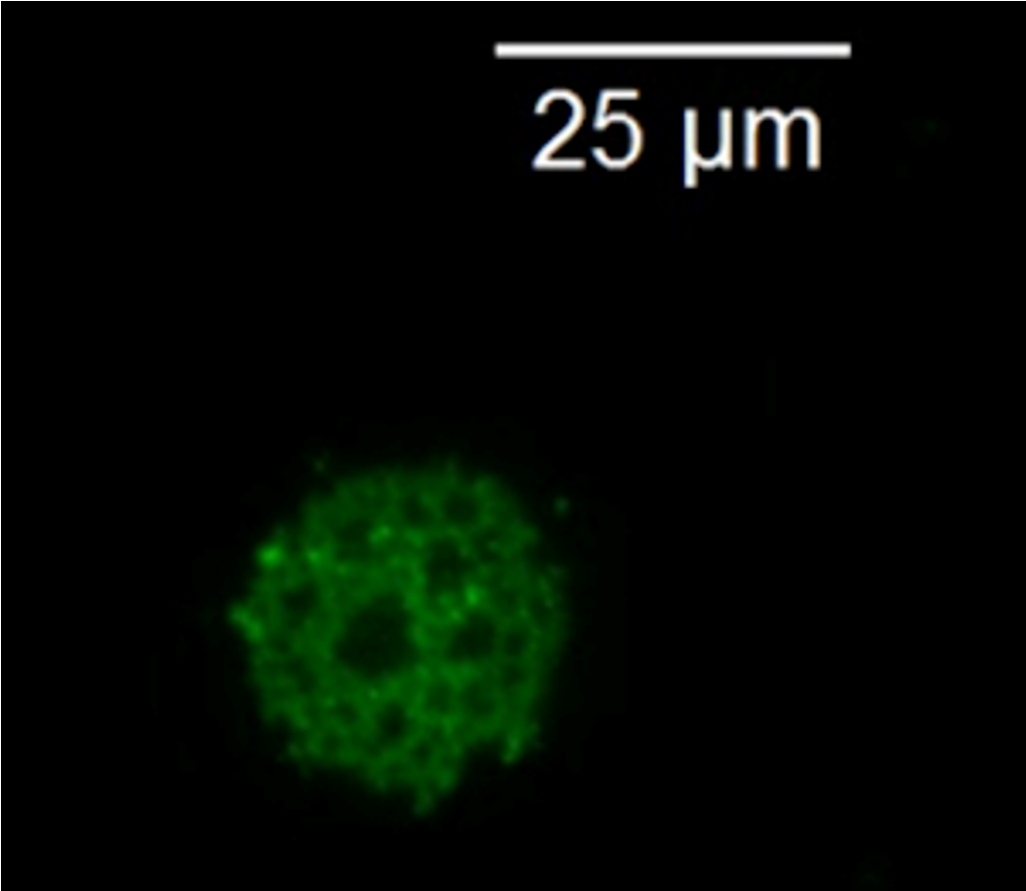
Cage-like microstructure obtained via sequential Ugi reactions.

After removing the solvent, the resulting structure could be easily identified using optical microscopy. They were cage-like microspheres with an average diameter corresponding to the diameter of droplets in the Pickering emulsion (Figure 2). The surface of the droplets was covered with a network of individual particles with holes of different diameter. It was likely that when deposited on the surface of the droplets, the particles stuck to each other, forming branched chains that gradually occupied all the available space.

This analysis confirmed incomplete coverage of the surface of droplets in the Pickering emulsions, which created a cage-like structure with a large distance between individual elements. At the same time, such a structure reliably protected against coalescence of individual droplets since Pickering emulsions obtained in this way were stable for a long time (more than one month). The formation of such structures opened up opportunities for the design of effective particle-based emulsifiers. The particles themselves consisted of 95–99% water. In addition, they did not completely cover the surface of the emulsion droplets. Therefore, the emulsifier consumption in terms of dry weight approached low-molecular-weight substances.

**Figure 3:**
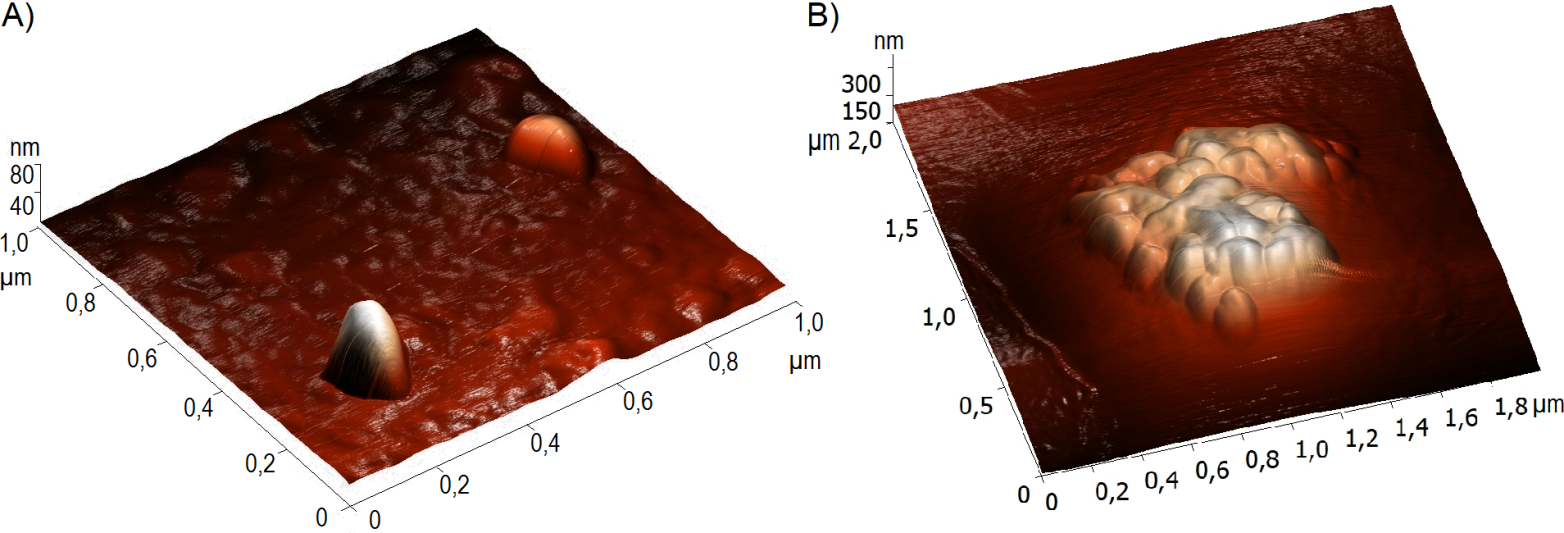
AFM images of A) single domains and B) cross-linked CMC/chitosan polymer particles.

We used AFM to identify the structural features of the original colloidal particles. For this, colloidal particles were adsorbed on the substrate surface and then analyzed without complete drying, which made it possible to reveal the initial microgel structure. Titration of CMC with hydrochloric acid, followed by treatment with a mixture of hexamethylene diisocyanide and formalin led to the formation of compact particles with an average diameter of 40–80 nm (Figure 3A). These particles were the main building blocks for the construction of cage-like structures. Following titration with a solution of low-molecular-weight chitosan and subsequent Ugi reaction, they formed agglomerates with a size of more than 500 nm that had a characteristic domain structure (Figure 3B).

These particles further interacted with each other on the surface of the droplets, forming a network. The repeated Ugi reaction resulted in the formation of a strong cage-like structure that was not destroyed upon removal of the solvent. We noticed a connection between the structure of the colloidal particles and their behavior at the interface. The developed surface of the particles, consisting of domains, promoted adhesion to each other and the formation of a network on the surface of the emulsion droplets. At the same time, the smooth surface of the particles contributed to the formation of a homogeneous monolayer at the phase interface.

**Figure 4:**
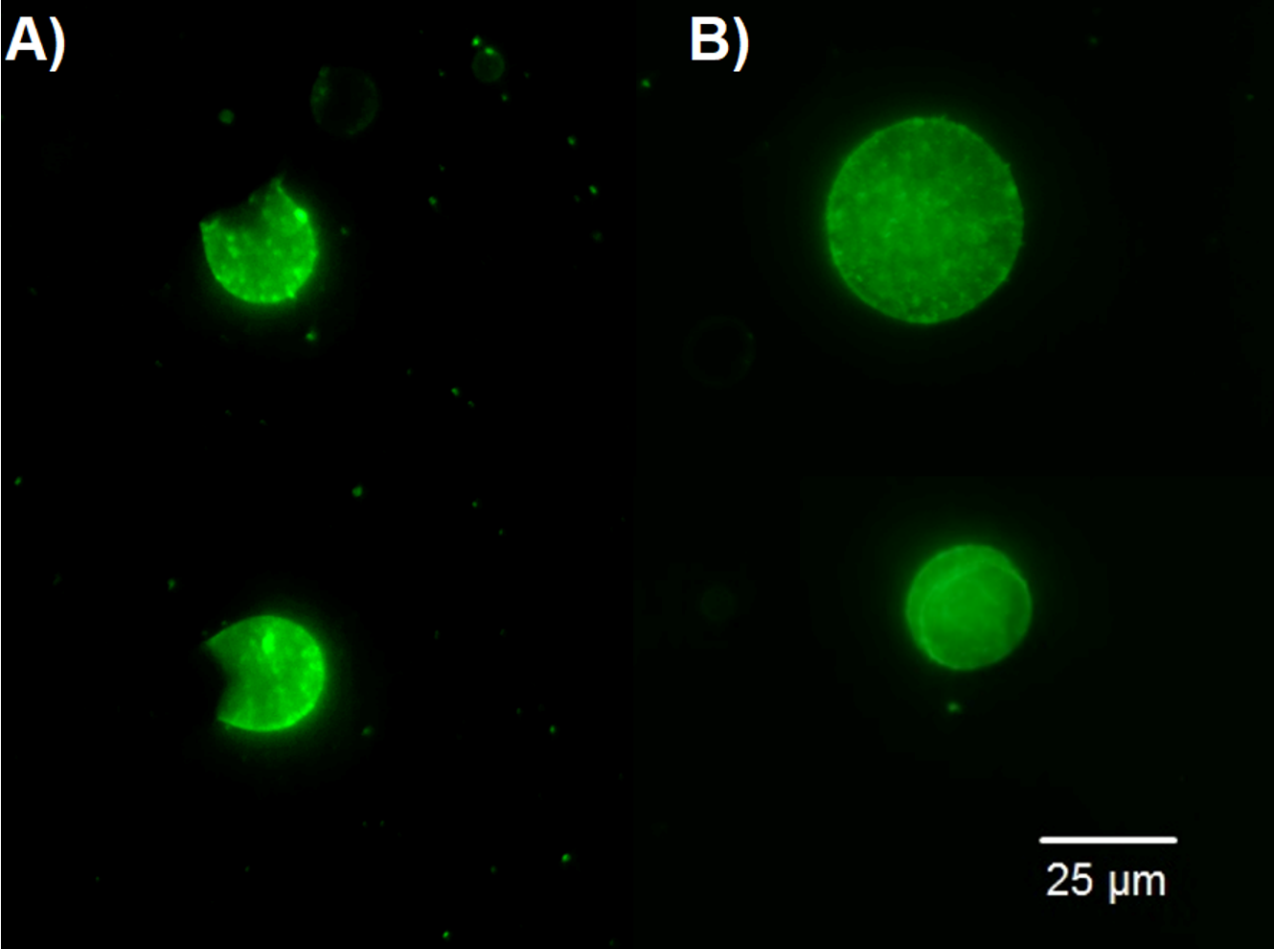
Variations of microstructures obtained via sequential Ugi reactions. A) Cap-like structures at 5 mol % cross-linking agent relative to chitosan at the second stage. B) Denser packing of particles at 20 mol % cross-linking density at the first stage.

To prove the influence of the structure of the colloidal particles on the formation of the surface in Pickering emulsions, we varied the conditions for their preparation. Specifically, we increased or decreased the cross-linking density using the Ugi reaction at the first (formation of colloidal particles) and second stages of the process (formation of a cage-like structure on the surface of emulsion droplets). Thus, when the amount of cross-linking agent at the second stage was halved to 5 mol % relative to chitosan, less durable microspheres were obtained. When the solvent was removed, they opened up, forming a cap-like structure (Figure 4A). An increase in the cross-linking density to 20 mol % at the first stage led to the formation of a denser packing of particles at the second stage of the process (Figure 4B). Supposedly, this was due to the large steric repulsion between the particles, which prevented the formation of chains on the surface of the emulsion droplets.

We also discovered that chitosan could be replaced by another polyamine in this synthesis, for example, polyvinylamine. In addition, the cellulose could be replaced by another polymer with carboxy groups, such as pectin. Thus, the scope of this synthesis can be significantly expanded in the future. These are the first examples showing how structure formation on the surface of emulsion droplets can be controlled by varying the conditions of the Ugi reaction. We are currently working to identify such dependencies and plan to publish the results of this work in the form of a full article.

## Conclusion

In summary, we have proposed a convenient approach for the synthesis of cage-like structures from carboxymethylcellulose and chitosan using the Ugi reaction. At the first stage, colloidal particles with a characteristic domain structure reminiscent of raspberries were created. At the second stage of the synthesis, a network of these particles was formed on the surface of the Pickering emulsion droplets. Removal of the solvent and redissolution in water led to the target structures. The Ugi reaction in water was an ideal choice because it was characterized by high reaction rates over a narrow pH value range. This allowed to control the process of cage-like-structure formation. Changing the conditions of the Ugi reaction made it possible to obtain various structures, including cage- and cap-like structures, as well as hollow microspheres. Due to the developed surface, these materials have potential to be used as catalysts and sorbents. Further, these materials may be obtained in significant quantities since the method for the production is simple and does not include stages that are difficult to scale up. Thus, the method we have proposed has good prospects for practical application.

## Supporting Information

File 1 Experimental data and optical microscopy data of the starting Pickering emulsions.

## Data Availability

The data that supports the findings of this study is available from the corresponding author upon reasonable request.
