# Supplementary material for: Cage-like microstructures via sequential Ugi reactions in aqueous emulsions
Source: Beilstein J Org Chem. 2024 Aug 22;20:2078–83. doi: 10.3762/bjoc.20.179 (PMC11346303; doi:10.3762/bjoc.20.179)
Supplement: File 1 — Experimental data and optical microscopy data of the starting Pickering emulsions. [file Beilstein_J_Org_Chem-20-2078-s001.pdf]

BEILSTEIN JOURNAL OF ORGANIC CHEMISTRY

Supporting Information

for

Cage-like microstructures via sequential Ugi reactions in aqueous emulsions

Rita S. Alqubelat, Yaroslava A. Menzorova and Maxim A. Mironov

Beilstein J. Org. Chem. **2024,** *20,* 2078–2083. doi:10.3762/bjoc.20.179

Experimental data and optical microscopy data of the starting Pickering emulsions

License and Terms: This is a supporting information file under the terms of the Creative Commons Attribution License (https://creativecommons.org/licenses/by/4.0). Please note that the reuse, redistribution and reproduction in particular requires that the author(s) and source are credited and that individual graphics may be subject to special legal provisions.
The license is subject to the *Beilstein Journal of Organic Chemistry* terms and conditions: (https://www.beilstein-journals.org/bjoc/terms)

Materials and Methods section

1. Materials

Carboxymethylcellulose (CMC, low viscosity polyanionic cellulose with degree of substitution 0.98, purity 95%, pH of 1% solution in water of 7.2 and apparent viscosity 19.4 mPa·s) was purchased from Henan Ocean Chemical Technology, China. Low molecular weight (17 kDa) chitosan with deacetylation ratio 0.95 and purity 98% was purchased from Heppe Medical Chitosan GmbH, Germany. Hexamethylene diisocyanide 95% was obtained using well known protocol [1]. Other materials included: dialysis membrane of molecular weights 8 kDa and 12 kDa (Orange Scientific, Belgium), fluoresceinamine, isomer I (Aldrich Chemistry, USA), *N*,*N*-dicyclohexylcabodiimide (Merck, Germany), organic reagents of analytical grade (Acros Organics, Belgium): toluene, acetonitrile, formaldehyde 35%, ethanol, dimethylformamide, hydrochloric acid, sodium hydroxide, phosphate buffer, acetic acid, deionized water.

2. Methods

2.1. Synthesis of fluorescein labeled CMC

A 20 mL solution of CMC in water with a concentration of 5% by weight is acidified with concentrated 10 N hydrochloric acid to pH 3 and then diluted with acetonitrile (50 mL). The precipitate that forms is washed with acetonitrile and redissolved in 20 mL of dimethylformamide. The solution of fluoresceinamine in dimethylformamide (3 mg in 1 mL) and an excess of dicyclohexylcarbodiimide (20 mg) is added to the resulting CMC solution in dimethylformamide. The mixture is left for a day in a dark place, then the resulting product is precipitated with acetonitrile. The resulting suspension is centrifuged at 500 rpm and then dried at 50 °C and the yield is determined after complete drying. Yield 850 mg (92%). For better dissolution in water, the drying stage can be skipped.

2.2. Preparation of CMC/chitosan microgels

CMC/chitosan microgels were prepared using a method we recently published [2]. Here we quote this work with minor changes:

A 250 mL of 0.1% CMC (in deionized water) is allowed to mix by stirring on a magnetic stirrer at a speed of 600 rpm for 30 min. After complete dissolution, 120 mL of 0.05% chitosan (in deionized water + 500 µL of acetic acid) is added dropwise to the solution and allowed to mix for 10 min at a speed of 600 rpm. The microgel suspension is acidified with 0.5 N solution of hydrochloric acid to pH 5.5, than hexamethylene diisocyanide (5 µL in 100 µL acetonitrile) and formaldehyde (10 µL in 125 µL of deionized water) are added to the mixture and allowed to mix for

90 min at an increased speed at 700 rpm. After stirring, the sample 100 mL is dialyzed using dialysis membrane of molecular weights 8 kDa against distilled water at pH 4. To determine the product yield and carry out physicochemical analyses, the solution after dialysis is diluted with a threefold amount of acetonitrile, the resulting precipitate is centrifuged and dried at 50 °C. To carry out further syntheses, the solution after dialysis is diluted to 0.3 g/L.

2.3. Preparation of cage-like microstructures

The solution 100 mL after dialysis with concentration of 0.3 g/L is mixed with toluene (the hydrophobic phase) in the ratio of 3:1. The content is emulsified using the IKA Ultra-Turrax T-25 homogenizer at a speed of 10,000 rpm for 5 min. Than, hexamethylene diisocyanide (2 µL in 100 µL acetonitrile) and formaldehyde (5 µL in 125 µL of deionized water) is added to the Pickering emulsion and allowed to mix for 90 min. A droplet of the emulsion is applied to a glass slide and dried at a temperature of 50 °C. Distilled water is then added and the sample is analyzed under a microscope.

2.4. Preparation of CMC/chitosan cap-like microstructures

The solution 100 mL after dialysis with concentration of 0.3 g/L is mixed with toluene (the hydrophobic phase) in the ratio of 3:1. The content is emulsified using the IKA Ultra-Turrax T-25 homogenizer at a speed of 10,000 rpm for 5 min. Than, hexamethylene diisocyanide (1 µL in 50 µL acetonitrile) and formaldehyde (2.5 µL in 60 µL of deionized water) is added to the Pickering emulsion and allowed to mix for 90 min. A droplet of the emulsion is applied to a glass slide and dried at a temperature of 50 °C. Distilled water is then added and the sample is analyzed under a microscope.

2.5. Preparation of pure CMC microgel sample for atomic force microscopy (AFM)

A 100 mL of 0.2% CMC (in deionized water) is allowed to mix by stirring on a magnetic stirrer at a speed of 600 rpm for 30 min. After complete dissolution, 60 mL of 0.01 N solution of hydrochloric acid is added dropwise to the solution and allowed to mix for 10 min at a speed of 600 rpm. Hexamethylene diisocyanide (10 µL in 200 µL acetonitrile) and formaldehyde (20 µL in 250 µL of deionized water) are added to the mixture and allowed to mix for 90 min at an increased speed at 700 rpm. After stirring, the sample 100 mL is dialyzed using dialysis membrane of molecular weights 8 kDa against distilled water at pH 4.

2.6. Sample preparation for AFM

The measurements are carried out on an NTEGRA Therma scanning probe microscope (NT-MDT, Zelenograd, Russia) using NSG01 silicon probes. The particle surface topography is measured in the semi-contact mode of AFM.

Particles are deposited on a cover slip, a 5 μL drop is placed on a substrate and the water is removed using filter paper and compressed air flow. The sample is washed in deionized water–a drop of deionized water is placed on the substrate in the area where the particles are located, after 1 minute the water is removed using filter paper and compressed air flow.

2.7. Sample preparation for optical microscopy of Pickering emulsion

Gels are studied using a laser scanning confocal microscope LSM-710 (Carl Zeiss, Germany) and fluorescent microscope Altami Lum1 Led. For this purpose, the droplet of Pickering emulsion is placed in a cup with a glass bottom (JetBiofill, China). Images are acquired in channel mode under excitation by a laser with a wavelength of 488 nm, with a confocal slice height of 9 μm. ImageJ program is used for image processing. Fluorescein-labeled CMC is used to obtain all pictures.

References

1. Ugi, I.; Fetzer, U.; Eholzer, U.; Knupfer, H.; Offermann, K. Isonitrile Syntheses. Angew. Chem., **1965**, 4, 472–484. doi:10.1002/anie.196504721

2. Mensah, E. O.; Alqubelat, R. S.; Menzorova, Y. A.; Minin, A. S.; Mironov, M. A. Colloids and Surfaces B: Biointerfaces, **2024**, 113827. doi:10.1016/j.colsurfb.2024.113827
